# Atrial Natriuretic Peptide Attenuates Colitis via Inhibition of the cGAS-STING Pathway in Colonic Epithelial Cells

**DOI:** 10.7150/ijbs.67356

**Published:** 2022-02-07

**Authors:** Chaoyue Chen, Ying Zhang, Meihui Tao, Xi Zhao, Qinyu Feng, Xiaoshang Fei, Yu Fu

**Affiliations:** Department of Gastroenterology, Union Hospital, Tongji Medical College, Huazhong University of Science and Technology, Wuhan 430022, China

**Keywords:** Atrial natriuretic peptide, STING pathway, Inflammatory bowel disease, Colonic epithelial cell, Gut barrier, Endoplasmic reticulum stress-induced autophagy

## Abstract

Atrial Natriuretic Peptide (ANP) has known anti-inflammatory effects. However, the role of ANP in Ulcerative colitis (UC) remains unclear. This study aimed to explore the expression and function of ANP in UC, and its potential regulatory role in the stimulator of interferon genes (STING) pathway. Human colon biopsy and serum samples were collected between September 2018 and December 2019 at Wuhan Union Hospital. Levels of ANP and its receptors and STING pathway components were detected in people with UC and mice with dextran sulfate sodium (DSS)-induced colitis. These mice and HT-29 cells were treated with ANP and an agonist of the STING pathway. The level of inflammation, STING pathway, gut barrier, and endoplasmic reticulum (ER) stress-induced autophagy were measured. We found that the levels of ANP and its receptor decreased and the STING pathway activated statistically in people with UC and the mouse model of colitis. ANP treatment attenuated DSS-induced colitis and inhibited STING pathway phosphorylation in colonic tissue and epithelial cells. An interaction between cGAS and NPR-A was verified. ANP repaired the gut barrier and inhibited ER stress-induced autophagy via the STING pathway. ANP may thus alter colonic barrier function and regulate ER stress-induced autophagy as a promising therapy for UC.

## Introduction

Inflammatory bowel disease (IBD) comprises several chronic idiopathic enteral diseases, including ulcerative colitis (UC) and Crohn's disease (CD). The pathogenesis of UC is still unknown, though it is related to disordered colonic mucosal immunity 1. Previous research has explored changes in cellular, humoral, and innate immunity in UC. UC is incurable, with current treatment options comprising medications and surgical interventions to reduce the burden of inflammation and induce and sustain disease remission. Biological agents can not only quickly alleviate the symptoms of UC but their long-term regular application can also promote and maintain mucosal membrane healing. However, due to the heterogeneity in response to treatment and adverse drug reactions among people with UC, it remains essential to develop new biological agents that target intestinal inflammation and promote mucosal membrane healing. We previously reported that interleukin 38 (IL-38) 2 and B cell activating factor (BAFF) 3 are involved in the regulation of colonic inflammation. However, current biological agents developed against various target molecules have large individual differences, high development costs, and unclear safety profiles, indicating a need to find novel therapeutic strategies for UC 4.

Atrial natriuretic peptide (ANP) is a hormone secreted by atrial muscle cells that exerts several physiological functions, such as regulating water and salt balance, blood pressure, and energy metabolism 5. ANP has been found in the lung, brain, liver, gastrointestinal tract, thymus, spleen, lymph nodes, tonsils, and other organs in humans 6. ANP and its receptors can also be detected in vascular smooth muscle cells, immune cells, and endothelial cells 7. Immunohistochemical studies have shown that there are ANP-expressing cells along the entire lamellar epithelium of the human gastrointestinal tract, including in the stomach, duodenum, jejunum, colon, and rectum 8. Recent studies have demonstrated that ANP exhibited endocrine, autocrine, or paracrine functions in immune regulation 9. ANP mainly exerts biological effects via receptors located on the plasma membrane of target cells, such as NPR-A and NPR-C 10-12. Previous studies have reported that the ANP/NPR-A axis exhibits anti-inflammatory activity. ANP has been shown to effectively reduce the expression of inducible nitrous oxide (iNOS) and tumor necrosis factor α (TNF-α) by activated macrophages 13. In NPR-A knockout mice, several pro-inflammatory cytokines were found to be increased in heart tissue, including TNF-α and interleukin 6 (IL-6) 14. Additionally, ANP is reported to have a more substantial anti-inflammatory effect than glucocorticoids in targeted tumor therapy^3^. Furthermore, it has been shown to significantly diminish the release of inflammatory factors in cytokine storms induced by bacterial infections and targeted drugs, and improve the survival rate in animal models 15.

Cyclic GMP-AMP synthase (cGAS)-stimulator of interferon genes (STING) is a recently discovered innate immune signaling pathway 16. It plays a defensive role by recognizing cyclic dinucleotides (CDNs) produced by bacteria and host DNA 17. When infection, stress, or cell damage occurs, cGAS recognizes pathogens in the cytoplasm and uses cytoplasmic adenosine triphosphate (ATP) and guanosine diphosphate (GTP) to generate the second messenger cyclic GMP-AMP (cGAMP) 18. cGAMP transduces the signal to the adaptor protein STING located on the endoplasmic reticulum (ER). STING recruits and activates TANK-binding kinase 1 (TBK1), which is followed by activation of the transcription factor interferon regulatory factor (IRF-3), thus promoting the release of inflammatory cytokines, including type I interferon (IFN) 18,19.

Emerging evidence has revealed that abnormal activation of the STING pathway is linked to metabolic disorders, infections, and inflammatory diseases 18,20,21. The cGAS-STING pathway is known to be involved in colonic inflammation and plays an essential role in maintaining immune homeostasis in the colonic mucosa 22. Previously published studies have shown some potential connections between ANP and the STING pathway; ANP binds to NPR-A and NPR-C to regulate the content of intracellular ATP and GTP 23, which are the substrates for cGAS catalysis of cGAMP formation 24. However, clear evidence for ANP regulation of the STING pathway remains lacking.

Fischer and colleagues previously revealed that STING is involved in tissue repair during acute colonic injury 25. However, Conway and colleagues indicated that STING signaling promotes TNF secretion and cell death in colonic organoids 26. UC is a typical inflammatory disease, in which colonic epithelial autophagy and ER stress defects, the disorder of intracellular environment all play key roles 27. STING is an ER resident protein with four N-terminal transmembrane domains. The interruption of ER homeostasis may affect STING signal transduction in colonic epithelial cells (CECs). Conversely, excessive activation of the STING pathway can disrupt calcium homeostasis and induce ER stress 28. Autophagy is an important antibacterial defense mechanism in the colonic mucosa during intracellular infection in various conditions of cellular stress, including ER stress. Autophagy is also the main process that is promoted by STING signal transduction 29. However, whether ANP and the STING pathway regulate colonic inflammation by changing the enteral barrier function and ER stress-induced autophagy remains to be determined.

In this study, we therefore aimed to investigate the role of ANP-mediated STING signaling in the pathogenesis of UC.

## Materials and Methods

### Human Samples

Colon biopsy samples for quantitative real-time polymerase chain reaction (qRT-PCR) were acquired from individuals with ulcerative colitis (UC) and control individuals. Focal colon biopsy samples for western blotting were acquired from patients with UC in activity and remission, and control individuals. Serum samples were collected from UC patients and control individuals. Specimens were gathered between September 2018 and December 2019 in the Department of Gastroenterology at the Union Hospital, Tongji Medical College, at the Huazhong University of Science and Technology.

A comprehensive diagnosis was made for those with UC based on clinical manifestations, imaging studies, endoscopic and pathological examinations, and exclusion of infectious enteritis and systemic diseases including cardiovascular diseases, which may influence the levels of serum ANP. Clinical disease activity was assessed using the Mayo Score Activity Index 30. Control individuals were those with hemorrhoids, polyps, or those undergoing health examinations with a regular colonoscopy.

### DSS-Induced Colitis Mouse Model and Treatments

Eight-week-old male C57BL/6 mice (22-24 g) were obtained from Beijing Weitong Lihua Animal Co. Mice were raised in specific pathogen-free conditions with sterile water and autoclaved food in an animal facility at Tongji Medical College. The mice were kept at room temperature (20-25°C) with a 12 h day/night cycle for a week before they were molded.

3% dextran sulfate sodium (DSS) (MP Biomedicals, USA) was administered in the drinking water of C57BL/6 mice for 7 days to induce acute colitis. Meanwhile, prazosin (0.2 mg in 400 μL phosphate buffered saline [PBS] per mouse; Xinyi Pharmacy, China), human ANP recombinant protein (2 μg in 400 μL PBS per mouse; Tocris Bioscience, England), STING pathway agonist DMXAA (0.1 mg in 400 μL PBS per mouse; Topscience, China), or an equal volume of PBS as a control was injected intraperitoneally into the mice at the same time daily. The DSS-induced disease course and dosing regimen for ANP and DMXAA are shown in Figures 2A and 7A. Weight change, fecal consistency, blood stool, and survival were recorded daily, and the disease activity index (DAI) was calculated by adding the values for percentage weight loss, fecal consistency, and blood stool 31. The mice were then euthanized, and the colon, spleen, and serum were collected for use in further experiments.

### Extraction of Primary CECs

Specimens (5 cm each) of murine colon were taken and the colonic segment was cut open lengthwise and rinsed thoroughly in autoclaved ice PBS (Gibco, USA) to remove residual blood stains on the surface, and the contents of the colonic lumen. The colonic segments were quickly cut on ice and placed in 40 ml of autoclaved ice PBS (Gibco) containing 1 mmol/L dithiothreitol (DTT; Gibco) and 1 mmol/L ethylenediaminetetraacetic acid (EDTA; Gibco), and incubated in water bath at 37°C for 20 min with continuous gentle shaking. The treated tissue suspension was filtered through a 70 μm filter screen, the undigested colonic tissues were removed, and the cell filtrate containing CECs was collected; this was performed twice. The resultant cell filtrate was centrifuged at 800 × g and 4°C for 3 min; the supernatant was carefully discarded, and 10 ml of 0.1% PBS was used to resuspend the cell sediment. Cells were gently inverted up and down to wash the cells. The cell suspension was centrifuged at 800 x g and 4°C for 5 min; centrifugation was repeated twice. Cells were resuspended with sterilized ice PBS, and the cell counting plate was prepared for counting.

### Histopathological Examinations

Small segments of the colons were fixed in 4% paraformaldehyde, embedded in paraffin, sectioned at 4 μm thickness, and dyed with hematoxylin and eosin (H&E). Histological activity index scores were used to assess colonic injury 32.

### Cell Culture and Treatment

The human colon cancer line HT-29 was obtained from the American Type Culture Collection (ATCC, American). HT-29 cells were cultured in RPMI-1640 (Gibco, USA) supplemented with 10% (v/v) fetal bovine serum (FBS; Gibco) and 1% (v/v) penicillin-streptomycin (Gibco) and maintained at 37°C and 5% CO_2_

STING pathway agonists DMXAA and human recombinant ANP were added to the cell culture medium of HT-29, where the concentration of DMXAA and ANP was 10 ug/ml, and the intervention time was 24 h.

### Immunohistochemical and Immunofluorescence Assays

Paraffin-embedded tissue blocks were dewaxed in xylene and rehydrated using a graded series of alcohol. Antigen recovery was performed in 10 mM citrate buffer. To quench endogenous peroxidase, slices were incubated for 15 min with 3% hydrogen peroxide. After washing with PBS three times, the slices were sealed with 10% unimmunized donkey serum for 30 min. After that, the slices were incubated with NPR-A rabbit polyclonal antibody (1:200; Affinity Biosciences, USA) at 4°C overnight. They were then washed with PBS and incubated with horseradish peroxidase-conjugated secondary antibody (1:200; Cell Signaling Technology, USA) at room temperature for 30 min. Then, the sections were 3, 3'-diaminobenzidine (DAB)-stained (AntGene Biotechnology, China) and visualized.

For the immunofluorescence assay in colon tissue, primary antibodies against E-cadherin, p-TBK1, and ZO-1 (all 1:200; Cell Signaling Technology, USA) were incubated with the colon sections at 4°C overnight, and relevant fluorescent secondary antibodies (Alexa Fluor 488 or Alexa Fluor 594, 1:200; AntGene Biotechnology, China) were added and incubated at 37°C for 1 h. After counterstaining the slices with 4',6-diamidino-2-phenylindole (DAPI; AntGene Biotechnology, China) for 15 min, we detected the location specific proteins via a laser scanning confocal microscope (Nikon, Japan).

For the immunofluorescence assay in HT-29 cells, cells were fixed with 4% paraformaldehyde; then the same steps were used as for colon tissue. Primary antibodies against E-cadherin, p-TBK1, P62, and cGAS (1:200; Cell Signaling Technology) and NPR-A (1:200; Santa Cruz Biotechnology, USA) were used.

### qRT-PCR

Total RNA was isolated using Trizol reagent (Takara, Japan) according to manufacturer's protocol. RNA concentration and quality were detected using a spectrophotometer (Thermo Fisher Scientific, USA). cDNA was obtained via reverse transcription using the PrimeScript™ RT kit (Perfect Real-Time, Takara, Japan). SYBR Premix Ex Taq™ (Tli RNaseH Plus, Takara, Japan) and the LightCycler^®^ 480 System (Roche, Switzerland) were used to detect gene expression. The conditions for gene amplification were as follows: pre-denaturation at 95°C for 10 min; 40 cycles of denaturation at 95°C for 30 s, annealing at 60°C for 1 min, and extension at 72°C for 30 s. Gene expression was standardized using mouse β-actin or human glyceraldehyde-3-phosphate dehydrogenase (GAPDH) as a reference. Relative levels of target genes were calculated using the 2^-ΔΔCt^ method. The primers used are shown in Table 1.

### Enzyme-Linked Immunosorbent Assay (ELISA)

Serum ANP levels were measured using an ANP Enzyme Immunoassay Kit (RayBiotech, USA), according to the manufacturer's protocol. Murine and human serum ANP concentrations were determined using a standard curve.

### Western Blot Assay

Total proteins were extracted from colonic tissues and HT-29 cells using radioimmunoprecipitation assay (RIPA) buffer (Beyotime Biotechnology, China) containing 1% protease and phosphatase inhibitors (MedChemExpress, USA). Total protein concentrations were quantified using a bicinchoninic acid (BCA) protein assay kit (Thermo Fisher Scientific). Protein lysates were separated using a 10% or 7.5% sodium dodecylsulfate polyacrylamide gel electrophoresis (SDS-PAGE) and then transferred to a polyvinylidene difluoride membrane. Membranes were closed using a closure buffer consisting of 5% bovine serum albumin (BSA; Antgene Biotechnology, China) in tris-buffered saline with 0.1% Tween-20 at room temperature for 1 h before incubation with primary and secondary antibodies (Antgene Biotechnology, China). Enhanced chemoluminescence western blotting substrate (ECL; Thermo Fisher Scientific, USA) was added to the membrane with target protein for 1 min. Exposure was controlled by an automatic exposure imaging system (Bio-Rad, USA). Primary antibodies against cGAS, STING, p-STING, TBK1, p-TBK1, IRF-3, p-IRF-3, LC3, P62, Beclin1, ATG12, BiP, eIF2α, P- eIF2α, JNK1, p-JNK (Cell Signaling Technology, USA), occludin (ABClonal Technology, China), and ZO-1 (Thermo Fisher Scientific, USA) were used.

### Co-Immunoprecipitation (Co-IP)

For the Co-IP assay, HT-29 cell lysates were incubated with anti-NPR-A antibody (1:100; Affinity Biosciences, USA) at 4°C overnight. Protein A/G agarose resin (Transgen, China) was added for 1h at 4°C. After washing with IP lysis buffer, immunoprecipitated complexes were eluted out from the Protein A/G resin. 1 × SDS loading buffer was added and the mixture was incubated at 95°C for 10 min to induce denaturation. Finally, protein samples were separated using SDS-PAGE and measured by western blotting using antibodies against NPR-A (1:1000; Affinity Biosciences, USA), cGAS (1:1000; Cell Signaling Technology, USA) and GAPDH (1:1000; Antgene Biotechnology, China).

### Assessment of Colonic Barrier Injury

Fluorescein isothiocyanate-dextran 4 kDa (FD4; Sigma, USA) was used to examine colonic permeability 33. Blood samples were collected 2 h after 750 mg/kg FD4 was fed to mice by intragastric administration. The serum was collected and separated to detect FD4. The concentration of serum FD4 was quantified using a fluorescence spectrophotometer (excitation 485 nm, emission 520nm; Thermo Fisher Scientific, USA).

### StubRFP-SensGFP-LC3 Lentiviral Transfection

HT-29 cells were grown to 50-70% confluency, then transfected with StubRFP***-***SensGFP***-***LC3 lentiviral particles (Genechem, China). After transfection, stable cell lines were obtained, and treated with DMXAA and/or ANP for 24 h. The cells were washed with PBS and fixed with 4% paraformaldehyde. A confocal laser scanning microscope (Olympus Corporation, Japan) was used to capture images of the red, green, and merged yellow channels to visualize LC3, representing autophagolysosomes and autophagosomes. The number of LC3 dots or 'puncta' per cell was manually counted.

### Statistical Analysis

Statistical analysis was performed using GraphPad Prism v8.0 software. Data were expressed as mean ± standard error (SEM). Analysis of variance (ANOVA) and Student's t tests were used to compare data. Spearman's correlation coefficient (r) was used to calculate the relationship between serum ANP level and clinical indicators. Kaplan-Meier methods were used for survival analysis. P ≤ 0.05 was considered statistically significant.

## Results

### Participants

In total, biopsy samples for PCR, biopsy samples for western blot, and blood samples for ELISA were separated into 3 groups (control, UC in activity, and UC in remission) and included in this study. Quantity and participants' clinical characteristics are shown in Table 2.

### The Expression of ANP and its Receptor Decreased in the Colon and Serum of Mice with Colitis and People with UC

Existing studies have shown that ANP can be detected in the lung, brain, liver, gastrointestinal tract, thymus, spleen, lymph nodes, tonsils, and other organs in humans 6. Here, we tested the expression of the ANP precursor gene, *NPPA* 34, and genes corresponding to the primary receptors for ANP, *NPR-A* and *NPR-C* 35, in the atrial liver, spleen, kidney, epityphlon, Peyer's patch, lymph gland, thymus, jejunum, ileum, and colon of untreated mice.

**Figure 1:**
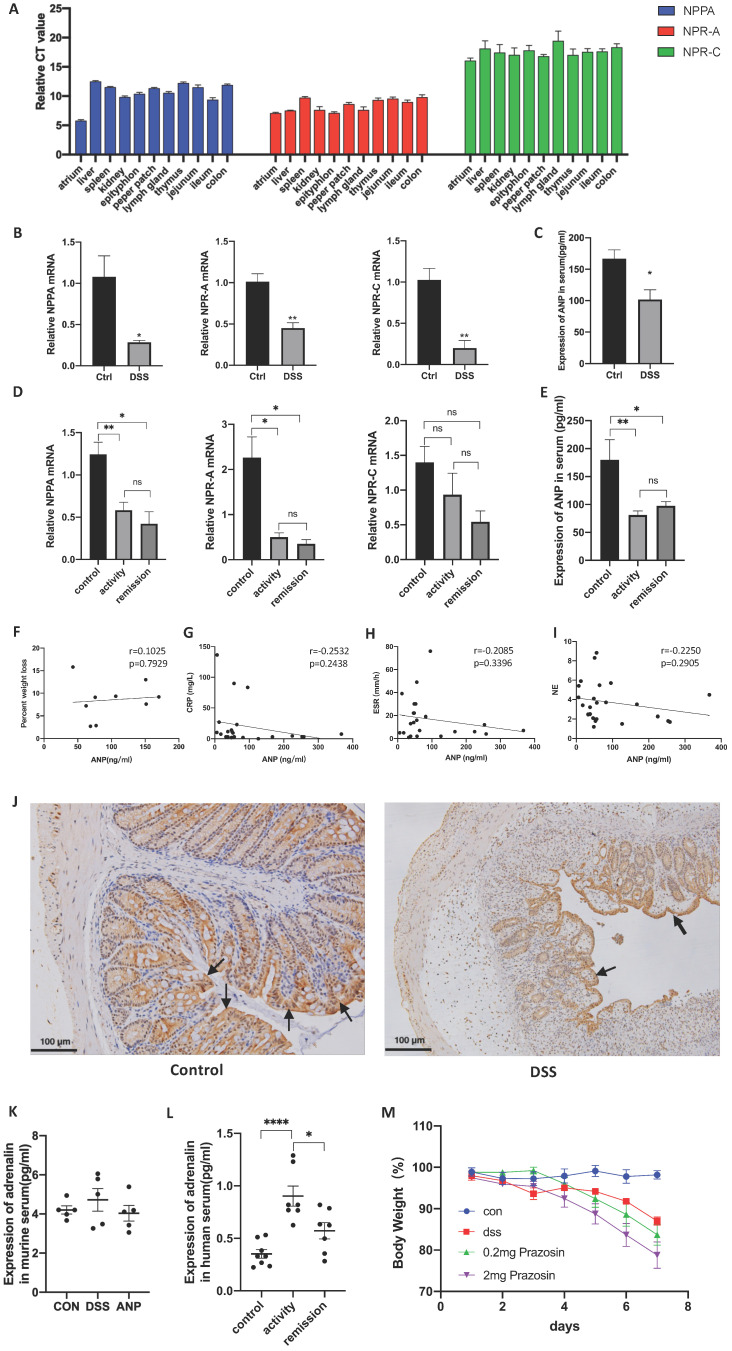
** Expression of atrial natriuretic peptide (ANP) and its receptor in colon and serum samples.** (A) Relative mRNA levels of the ANP precursor (NPPA) and ANP receptors (NPR-A and NPR-C) in different organs of wild-type mice as determined by quantitative real-time polymerase chain reaction (qRT-PCR; n = 5 for each group). The Y axis represents the expression level difference between the cycle threshold (CT) value of the target gene and the β-actin, and the smaller the Y axis value, the higher the gene expression. (B) Mice were treated with 3% dextran sulfate sodium (DSS) in drinking water for 7 days. Relative expression of *NPPA, NPR-A*, and *NPR-C* in the colon of control and DSS-treated groups was detected by qRT-PCR. Results were normalized against the β-actin gene. (C) Level of ANP was determined in the serum of a DSS-induced colitis and control mice using an enzyme-linked immunosorbent assay (ELISA). (D) Relative expression of *NPPA*, *NPR-A*, and *NPR-C* in the colon of people with UC in activity (n = 15), UC in remission (n = 9) and control individuals (n = 45) was determined using RT-qPCR. (E) Level of ANP was detected in serum from UC patients with active disease (n = 12) and remission (n = 11) and control individuals (n = 27) using ELISA. (F) Correlation between murine serum ANP and percent weight loss. (G-I) Correlation between serum ANP and C-reactive protein (CRP) level, erythrocyte sedimentation rate (ESR) and neutrophilicgranulocyte (NE) level. (J) *NPR-A* expression was detected by immunohistochemical analysis in murine colonic tissue. (K) The level of adrenaline was determined using ELISA with the treatment of DSS and ANP or not. (L) The level of adrenaline was detected in serum from people with UC or controls using ELISA. (M) Mice with a DSS-induced experimental model of colitis (n = 6 in each group) were intraperitoneally injected with prazosin or PBS. Bodyweight curves were recorded. Statistical analysis was performed using T-tests: NS (not significant), P > 0.05; *P < 0.05; **P < 0.01; and ****P < 0.0001 are shown on the figure.

As shown in Figure 1A, *NPPA*, *NPR-A* and *NPR-C* were all expressed in these organs at different levels, and the expression of NPR-A was significantly higher than NPR-C, indicating that NPR-A is the main receptor. Next, we detected *NPPA*, *NPR-A*, and *NPR-C* expression in the DSS-induced colitis mouse model and people with UC, and found that the mRNA levels corresponding to all three genes were decreased in colonic tissues of the DSS-induced colitis mouse model (Figure 1B), and in colonic tissue of people with UC (Figure 1D). *ANP* expression in the serum was also reduced in the DSS-induced colitis mouse model and people with UC compared to control group (Figure 1C, E). In mice with colitis, the serum *ANP* expression level was not found to be related to the degree of weight loss (Figure 1F). No statistically significant associations between serum *ANP* expression and C-reactive protein (CRP) level, erythrocyte sedimentation rate (ESR), or neutrophilicgranulocyte (NE) level was observed in people with UC (Figure 1G-I). We also examined the location of *NPR-A* expression in murine colon by immunohistochemical analysis. Typical images are shown in Figure 1J, which show that *NPR-A^+^* cells were found mainly in the colonic epithelial cells and were markedly decreased in the DSS-induced mouse model of colitis compared to control group.

Previous research has reported that ANP inhibits the secretion of inflammatory factors by reducing the level of serum adrenaline. The level of serum adrenaline in people with UC was higher than in healthy people in our study (Figure 1K). However, the serum adrenaline level in the DSS-induced mouse model of colitis was not dramatically increased compared with the healthy group, and the level in the mice with colitis did not decrease to a significant extent after intraperitoneal injection with ANP (Figure 1L). To clarify whether adrenaline regulates colonic immunity in the mice with colitis, we used the adrenergic receptor blocker prazosin as an intervention, which did not alleviate colonic inflammation in these mice (Figure 1M).

### ANP Ameliorates DSS-Induced Colitis in Mice

**Figure 2:**
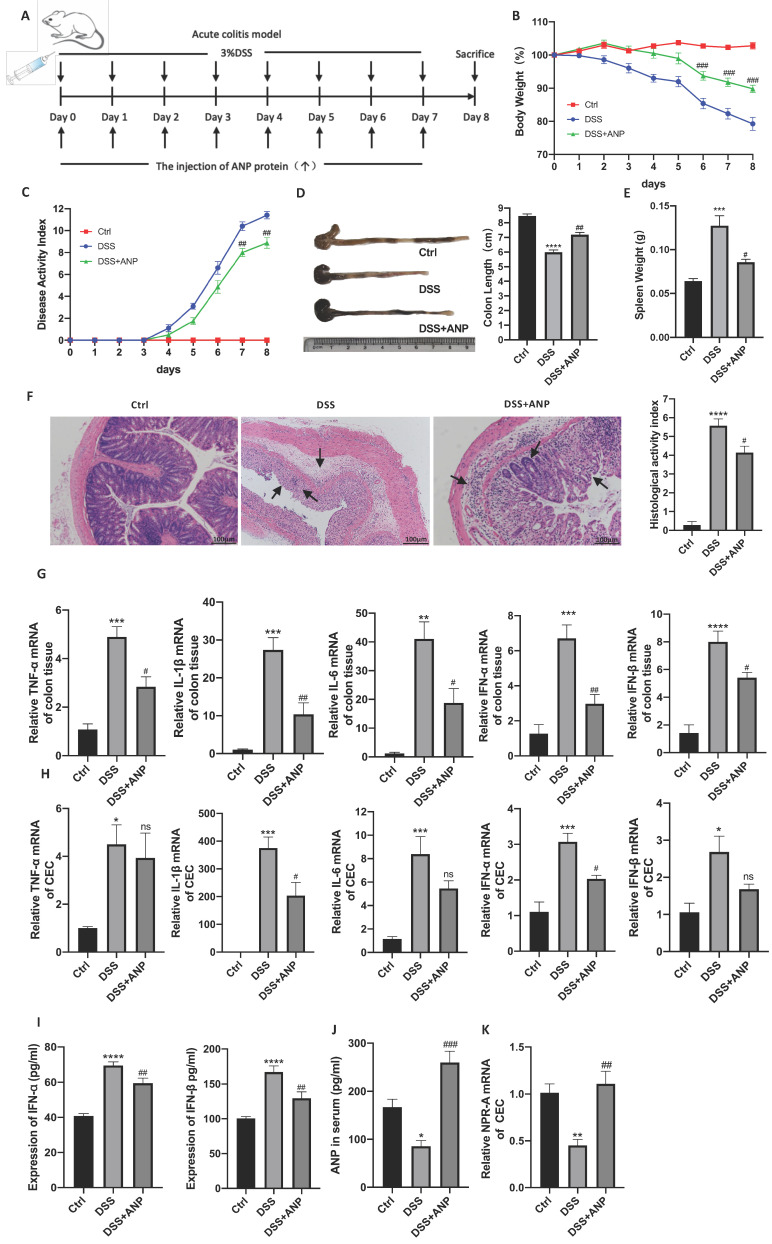
** Atrial natriuretic peptide (ANP) ameliorates dextran sulfate sodium (DSS)-induced colitis in mice.** Mice with DSS-induced experimental colitis (n = 6-9 in each group) were intraperitoneally injected with ANP recombinant protein or phosphate buffered saline (PBS). Mice were sacrificed, and samples were collected on the 8th day. (A) Detailed method and dosing regimen for DSS and ANP. (B) Bodyweight curves. (C) Disease activity index (DAI) scores. (D) Typical image of a colon, from which length was measured. (E) Spleen weight. (F) Hematoxylin and eosin (H&E) staining of murine colonic slices and the histological activity index score for colon tissue. (G-H) Relative expression of tumor necrosis factor α (*TNF-α*), Interleukin 1β (*IL-1β*), *IL-6*, interferon α (*IFN-α*), and *IFN-β* in colon tissue and colonic epithelial cells (CECs) were determined using quantitative real-time polymerase chain reaction (RT-PCR). (I) Levels of IFN-α and IFN-β were determined in serum samples using an enzyme-linked immunosorbent assay (ELISA). (J) Levels of ANP were determined in the serum by ELISA. (K) Relative expression of *NPR-A* in colon tissue and CECs was determined by qRT-PCR. Data are shown as mean ± standard error (SEM). n = 6-9 in each group. Statistical significance was calculated using one way analysis of variance (ANOVA). (Compared with the control group, *P < 0.05; ***P < 0.001; ****P < 0.0001. Compared with the DSS-treated group, NS, P > 0.05; #P < 0.05; ##P < 0.01; ###P < 0.001).

Although our results showed that *ANP* expression was decreased in colitis, the role of ANP in UC pathogenesis was unclear. Therefore, we used ANP recombinant protein to treat DSS-induced colitis in mice. A detailed method and dose schedule for DSS and ANP is presented in Figure 2A. Compared with the mice treated with PBS, those injected with ANP had significantly decreased weight loss, colon shortening, spleen weight, and DAI results (Figure 2B-E). H&E staining showed substantial destruction in colonic structure in mice with colitis who received PBS, as indicated by cavities where inflammatory cells infiltrated and disappeared into the lamina propria. ANP treatment significantly improved structural injury and colonic inflammation (Figure 2F). These results indicated that ANP efficiently alleviated the severity of colonic inflammation in mice with colitis.

To explore whether ANP could ameliorate colonic inflammation by altering colonic cytokine production, we detected levels of inflammatory cytokines (TNF-α, IL-1β, IL-6) and cytokines related to the STING pathway (IFN-α and IFN-β) in colonic tissues using qRT-PCR. As shown in Figure 2G, ANP downregulated TNF-α, IL-1β, IL-6, IFN-α, and IFN-β mRNA levels in the colonic tissue in the mouse model of colitis (P < 0.05). To explore whether ANP played an anti-inflammatory role in CECs, we extracted murine original CECs and detected TNF-α, IL-1β, IFN-α, and IFN-β levels. The results showed that IL-1β and IFN-α levels were significantly suppressed by ANP treatment compared with the only DSS group, while changes in TNF-α and IFN-β were negligible (Figure 2H). Serum IFN-α and IFN-β levels in mice with colitis were reduced in the ANP-treated group (Figure 2I). ANP treatment significantly improved the level of serum ANP and the mRNA expression of NPR-A in the CECs of DSS mice (Figure 2J-K). These results showed that ANP treatment suppressed the production of inflammatory cytokines, including those related to the STING pathway, in colonic tissue and CECs in mice with DSS-induced colitis. 

### The STING Pathway is Activated in the Colon of People with UC

**Figure 3:**
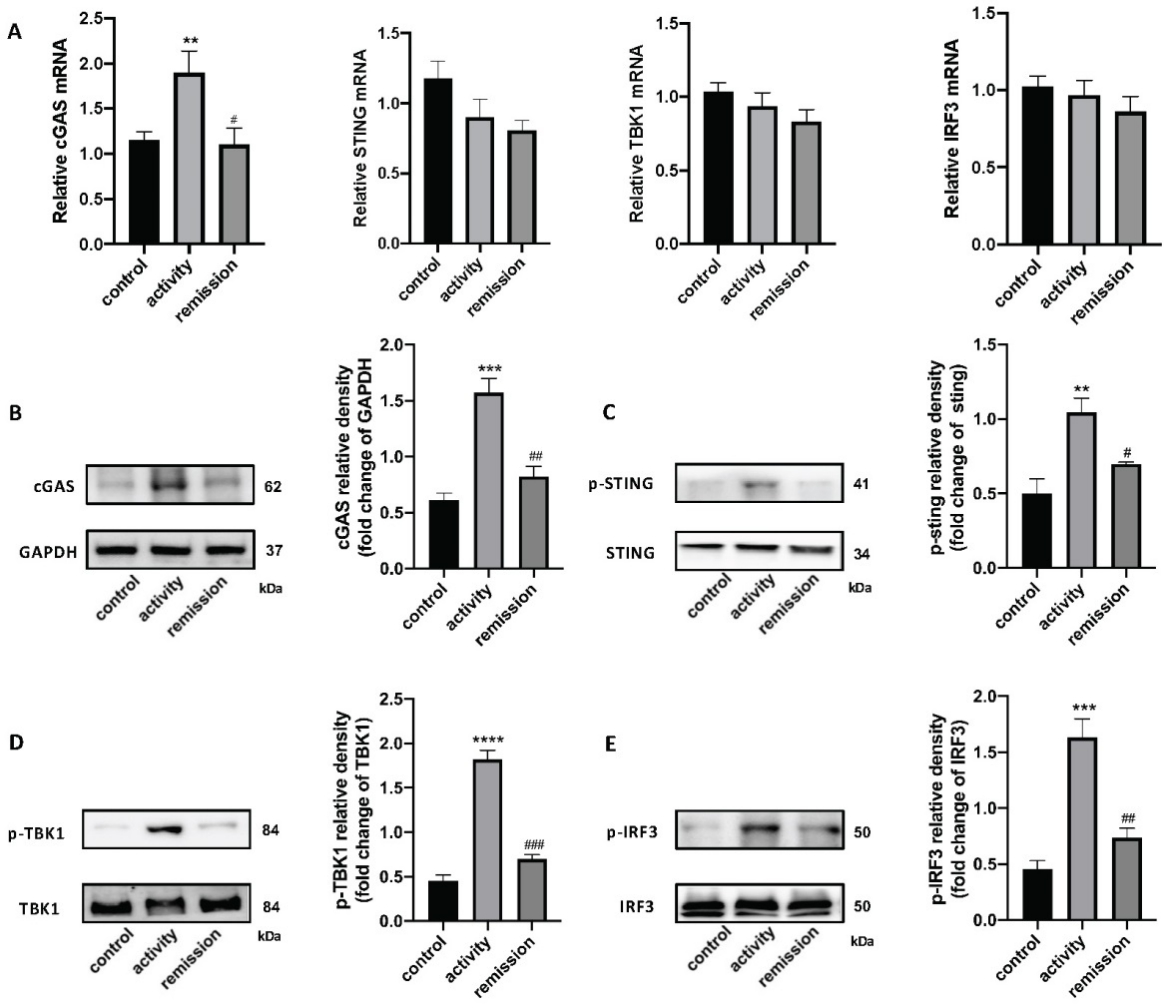
** Stimulator of interferon genes (STING) pathway expression in colonic tissue of people with UC.** (A) Relative expression of STING pathway related genes, including cGAS, STING, TBK1, and IRF3, in the colon of with UC patients in activity (n = 15), UC patients in remission (n = 9) and control individuals (n = 45), was tested using quantitative real-time polymerase chain reaction (qRT-PCR). Results were normalized against the glyceraldehyde-3-phosphate dehydrogenase (*GAPDH*) gene. (B-E) Expression levels of STING pathway related proteins in colonic tissue as determined by western blotting (n = 6 in each group). Representative blots are shown, and quantitative density data are shown as the intensity ratio of the target protein to relevant controls: (B) cGAS/GAPDH; (C) p-STING/STING; (D) p-TBK1/TBK1; (E) p-IRF3/IRF3. Statistical analysis was performed using T-tests. NS, P > 0.05; *P < 0.05; and **P < 0.01.

qRT-PCR and western blotting were used to detect the STING pathway in the colonic tissue of people with UC. *cGAS* mRNA levels were increased in UC patients in activity and unchanged in UC patients in remission, while *STING*, *TBK1*, and *IRF3* levels were unchanged (Figure 3A). We also tested STING pathway protein levels and their phosphorylation by western blotting, which demonstrated that the level of cGAS protein was increased in UC patients in activity compared with UC patients in remission and control individuals. The phosphorylation levels of STING, TBK1, and IRF3 were obviously increased in activity group compared with remission and control group (Figure 3B-E). These results indicate that the STING pathway was activated in the colons of people with UC in activity.

### ANP Promotes cGAS/NPR-A complex formation

**Figure 4:**
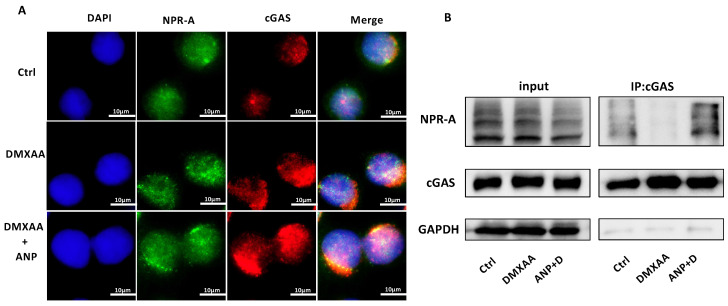
** Atrial natriuretic peptide (ANP) promotes cGAS/NPR-A complex formation.** HT-29 cells were treated with DMXAA or ANP 24 h in advance (n = 5 for each group). (A) Localization of NPR-A (green fluorescence) and cGAS (red fluorescence) within HT-29 cells, as evaluated by immunofluorescence. (B) HT-29 cells were subjected to immunoprecipitation with cGAS or NPR-A antibodies, followed by western blotting using the indicated antibodies.

Both *NPR-A* and *cGAS* were found to be expressed in HT-29 cells. The expression of *cGAS* increased with the addition of DMXAA, and the co-localization of cGAS and NPR-A proteins were observed with combined DMXAA and ANP treatment (Figure 4A). We used a Co-immunoprecipitation assay to confirm the role of ANP in promoting the formation of a cGAS/NPR-A complex (Figure 4B). The results showed clear bands in both the input group and the immunoprecipitation group, confirming the presence of a cGAS/NPR-A complex. In addition, the amount of this complex increased significantly when DMXAA and ANP were administered simultaneously; when DMXAA was administered alone, levels of the complex decreased. These data indicate that the levels of the cGAS/NPR-A complex were influenced by ANP.

### ANP Inhibits STING Pathway Activation and Repairs Gut Barrier Damage in CECs

**Figure 5:**
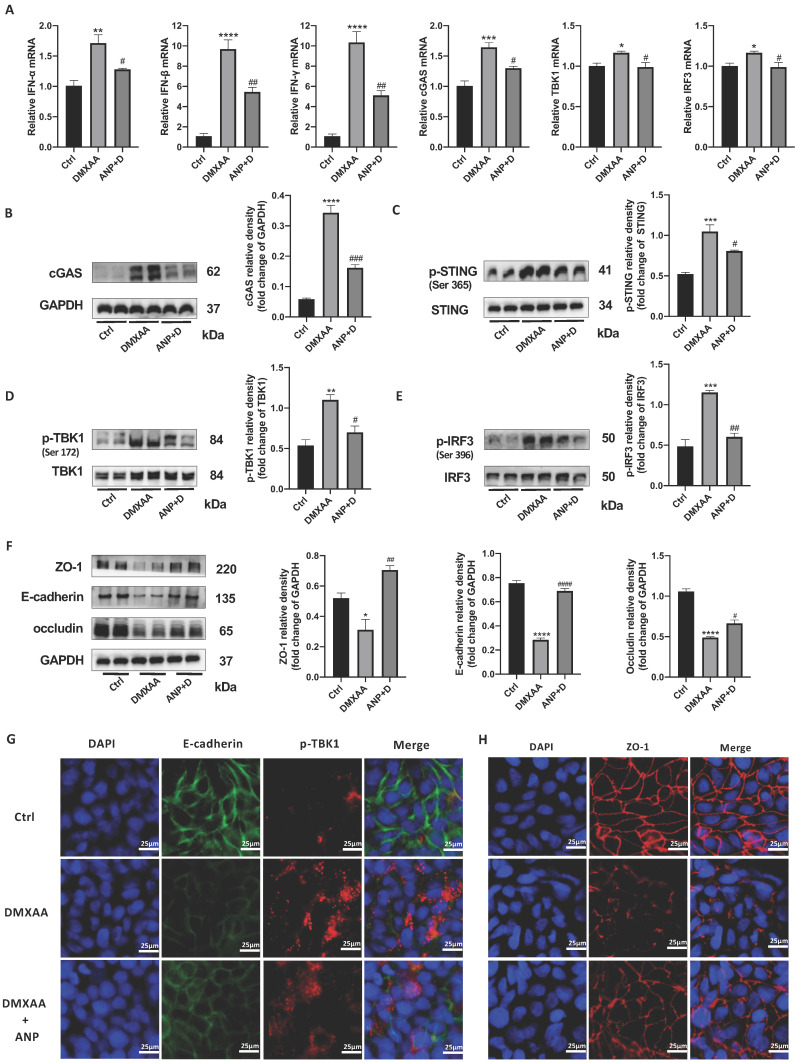
** Atrial natriuretic peptide (ANP) inhibits stimulator of interferon genes (STING) pathway activation and repairs gut barrier damage in colonic epithelial cells (CECs).** HT-29 cells were treated with DMXAA or ANP 24 h in advance (n = 5 for each group). (A) Relative expression of interferon α (IFN-α), IFN-β, IFN-γ, cGAS, TBK1, and IRF3 in HT-29 cells was tested using quantitative real-time polymerase chain reaction (qRT-PCR. Results were normalized against the glyceraldehyde-3-phosphate dehydrogenase (*GAPDH*) gene. (B-E) Expression levels of STING pathway related proteins in colon tissue, as identified by western blotting. Representative blots are shown, and quantitative density data are shown as the intensity ratio of the target protein to relevant controls: cGAS/GAPDH; p-STING/STING; p-TBK1/TBK1; and p-IRF3/IRF3. (F) Protein levels of ZO-1, E-cadherin, and occludin in HT-29 cells, as determined by western blotting (n = 5 for each group). Results were normalized against GAPDH. (G) Localization of E-cadherin (green fluorescence) and p-TBK1 (red fluorescence) within HT-29 cells, evaluated by immunofluorescence. (H) Localization of ZO-1 (red fluorescence) within HT-29 by immunofluorescence.

From our results, we found that NPR-A was mainly expressed in the colonic epithelium, ANP reduced the expression of inflammatory factors in primary CECs, and ANP repaired the colonic epithelial barrier in mice via the STING pathway. We therefore inferred that ANP regulates the STING pathway in CECs. To test this, we treated HT-29 with DMXAA and ANP, and detected mRNA levels for *IFN-α/β* and STING pathway-related genes in cell lysates. The results showed that DMXAA promoted the transcription of *IFN-α/β* and *cGAS* (Figure 5A). Then, we detected the levels of STING pathway-related proteins using western blotting; we found that cGAS and phosphorylated STING, TBK1, and IRF3 levels were increased after treatment with DMXAA, and decreased after treatment with ANP compared with DMXAA treatment (Figure 5B-E). Using immunofluorescence, we found that the level of phosphorylated TBK1 in the DXMAA treated group was increased, and ANP reduced the level of phosphorylated TBK1 (Figure 5G).

Moreover, the tight junction proteins ZO-1 and occludin and the epithelial cell marker protein E-cadherin were detected, and we also found that ANP promoted the synthesis of these proteins following DMXAA destruction of the colonic barrier (Figure 5F). Our immunofluorescence results for ZO-1 also showed similar results (Figure 5H).

### ANP Inhibits ER Stress-Induced Autophagy in CECs via STING Pathway Activation

**Figure 6:**
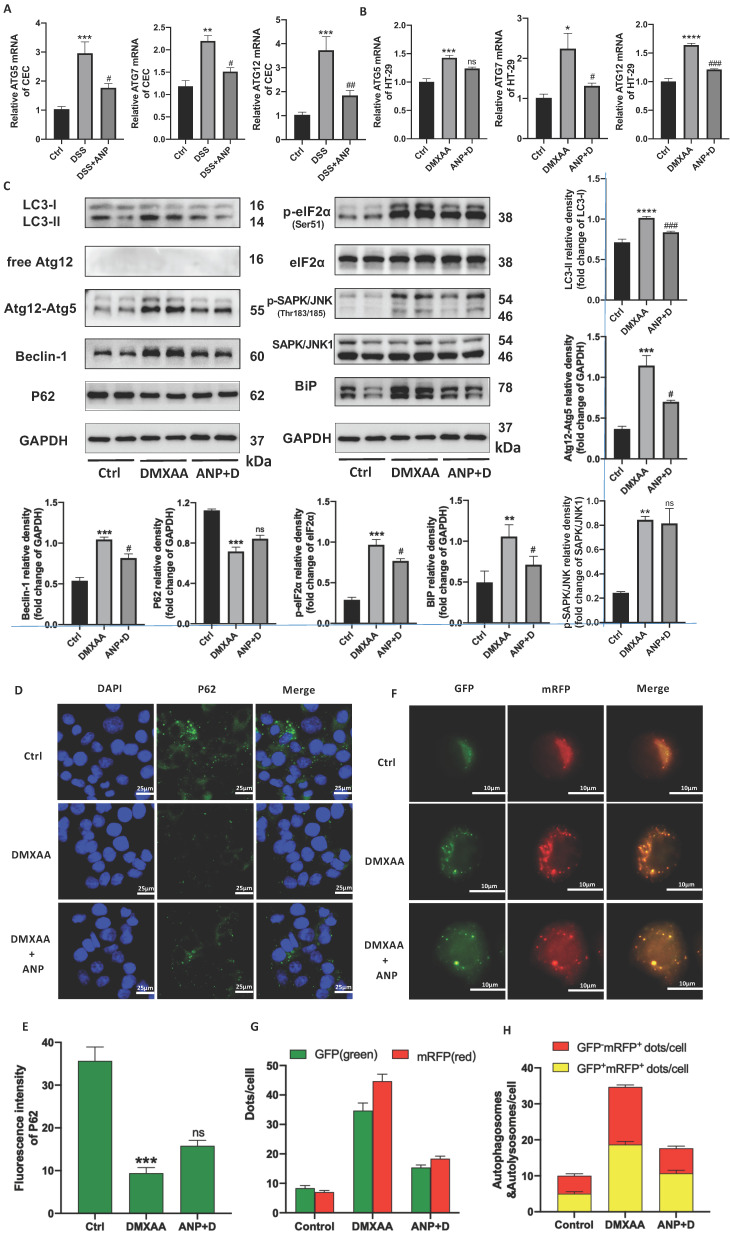
** Atrial natriuretic peptide (ANP) inhibits endoplasmic reticulum (ER) stress-induced autophagy in colonic epithelial cells (CECs) via stimulator of interferon genes (STING) pathway activation.** HT-29 were treated with DMXAA or ANP 24 h in advance (n = 5 for each group). (A-B) Relative expression of *Atg5, Atg7,* and* Atg12* in primary CECs extracted from mice and in HT-29 cells. Results were normalized against the β-actin/ glyceraldehyde-3-phosphate dehydrogenase (*GAPDH*) genes. (C) Expression levels of autophagy-related (LC3, free Atg12, Atg12-Atg5, Beclin1, P62) and ER stress-related (eIF2α, SAPK/JNK, BiP) proteins in HT-29 were identified using western blotting (n = 5 for each group). Representative blots are shown, and quantitative density data are shown as the intensity ratio of the target protein to relevant controls: LC3-II/LC3-I, Atg12-Atg5/GAPDH, Beclin1/GAPDH, P62/GAPDH, p-eIF2α/eIF2α, p- SAPK/JNK/SAPK/JNK, and Bip/GAPDH. (D-E) Localization of P62 (green fluorescence) in HT-29 cells, as evaluated by immunofluorescence. (F-H) The accumulation of StubRFP-SensGFP-LC3 in HT-29 cells treated with DMXAA or ANP was observed using confocal micrographs. Red arrows indicate autophagosomes and yellow arrows indicate autolysosomes.

In light of previous studies showing that the STING pathway is closely related to ER stress and autophagy, we detected autophagy and ER stress-related indicators in murine primary CECs. We found that *ATG5/7/12* mRNA levels increased in mice with colitis, and that ANP prevented this increase (Figure 6A). Consistent changes were also found in HT-29 cells treated with DMXAA and ANP (Fig 6B). In a western blot analysis to detect the expression of autophagy-related proteins (LC3, Atg12, Beclin1, and P62) it was found that, after DMXAA stimulation, the STING pathway proteins LC3-II/I increased, along with the expression of the ATG12-ATG5 complex and Beclin1, while the expression of P62 decreased. This suggests that the activation of the STING pathway was accompanied by activation of autophagy in CECs. After the addition of ANP, STING pathway-induced autophagic activation was inhibited (Figure 6C).

Western blotting was also used to measure the levels of phosphorylated and unphosphorylated BiP, eIF2α, and SAPK/JNK; eIF2α, SAPK/JNK, and BiP phosphorylation increased in mice treated with DMXAA, indicating that STING pathway activation promoted ER stress in CECs. This effect was inhibited with ANP treatment (Figure 6C).

The P62 protein was labeled in HT-29 cells using immunofluorescence, and its pattern of expression was consistent with that observed in our western blot (Figure 6D, E). HT-29 cells were transfected with StubRFP-SensGFP-LC3 lentiviral particles and treated with DMXAA and ANP. The red spots represent autophagic lysosomes, and the yellow spots stand for autophagosome. Fluorescence microscopy showed that the number of red (GFP^-^mRFP^+^) and yellow spots (GFP^+^mRFP^+^) increased in the DMXAA-treated group, and the number of both types of spot decreased in the group treated with ANP and DMXAA, suggesting that ANP inhibits STING pathway-induced autophagy (Figure 6F, G). After merge, the number of red and yellow spots increased in the DMXAA-treated group, and decreased in the group treated with ANP and DMXAA, indicating activation and inhibition of autophagy, respectively (Figure 6F, H).

### ANP Inhibits STING Pathway Activation and Repairs Gut Barrier Damage in a Mouse Model of Colitis

**Figure 7:**
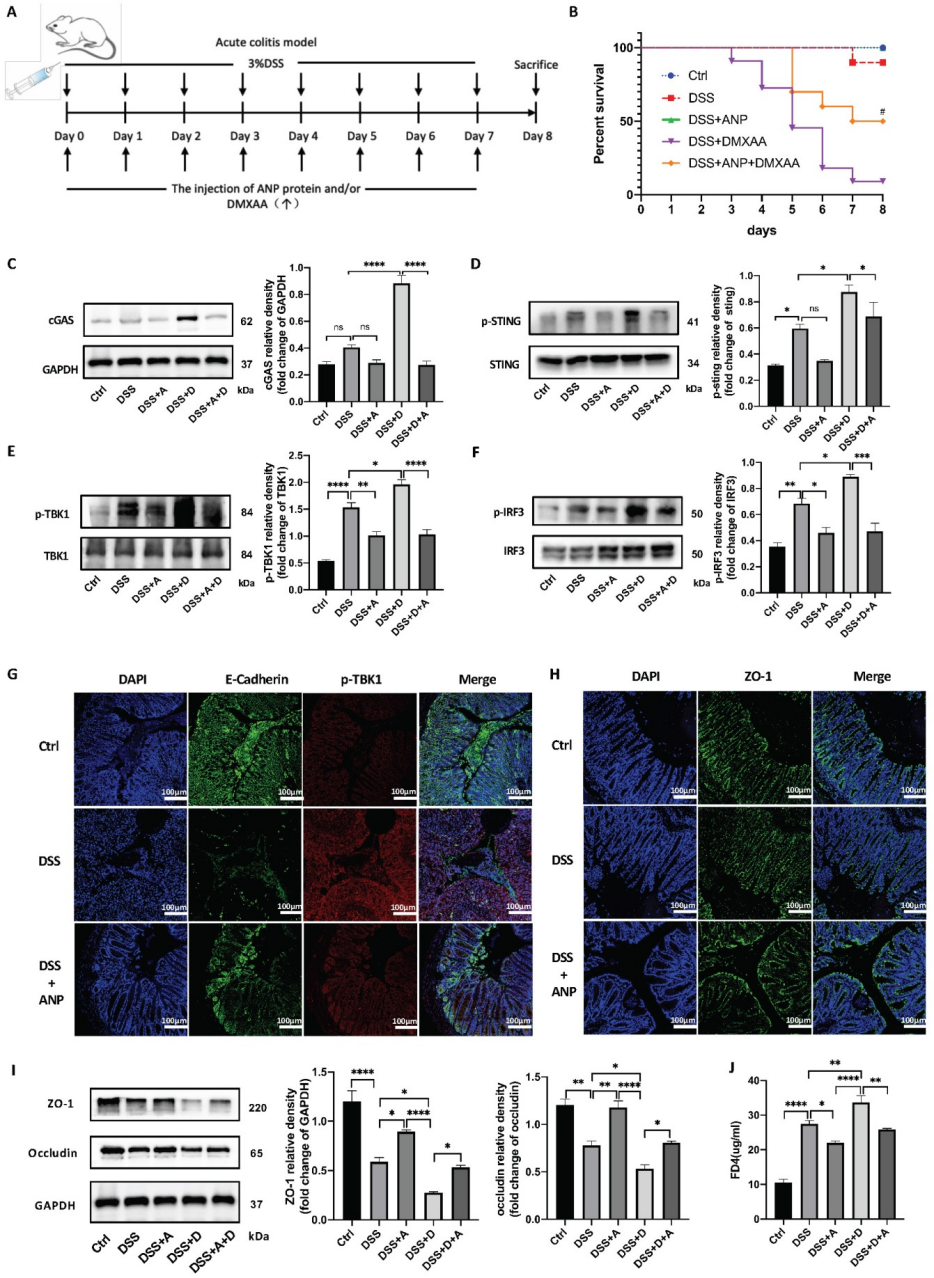
** Atrial natriuretic peptide (ANP) inhibits stimulator of interferon genes (STING) pathway activation and repairs gut barrier damage in a dextran sulfate sodium (DSS)-induced colitis mouse model.** Mice with DSS-induced experimental colitis (n = 12 in each group) were intraperitoneally injected with ANP recombinant protein (2 μg in 400μL phosphate buffered saline [PBS] per mouse), DMXAA (0.1 mg in 400μL PBS per mouse) or 400μL PBS. Mice were sacrificed, and samples were collected on the 8th day. (A) Detailed method and dosing regimen for DSS, ANP and DMXAA. (B) Survival rates of mice with DSS-induced colitis. (C-F) Levels of STING pathway related proteins in colonic tissue, as identified by western blotting (n = 3 in each group). Representative blots are shown, and quantitative density data are shown as the intensity ratio of the target protein to relevant controls: cGAS/GAPDH; p-STING/STING; p-TBK1/TBK1; p-IRF3/IRF3. (G) Localization of E-cadherin (green fluorescence) and p-TBK1 (red fluorescence) within the colonic mucosa, as evaluated by immunofluorescence. (H) Localization of ZO-1 (green fluorescence) within colonic mucosa as evaluated by immunofluorescence. (I) Levels of ZO-1 and occludin in colonic tissue, as determined by western blotting (n = 5 in each group). Results were normalized against glyceraldehyde-3-phosphate dehydrogenase (GAPDH). (J) The levels of serum FD4 in different groups, reflecting colonic permeability. Statistical analysis was performed using one way analysis of variance (ANOVA; *P < 0.05, **P < 0.01, ***P < 0.001, ****P < 0.0001. Compared with group treated with DSS and DMXAA, #P < 0.05).

Previous studies have shown that DMXAA activates the STING pathway and aggravates DSS-induced experimental colitis 36. To investigate whether ANP ameliorates DSS-induced experimental colitis via the STING pathway, we administered ANP and DMXAA separately or in combination to mice with colitis. The detailed procedure and dose regimen of DSS, ANP, and DMXAA are shown in Figure 7A. DMXAA was found to significantly reduce the survival rate in the mice, while ANP rescued survival to a significant degree (Figure 7B). To confirm whether ANP ameliorates colitis via the STING pathway, we detected the expression levels of STING pathway-related proteins using western blotting (Figure 7C-F). The cGAS protein levels were increased after treatment with DMXAA in the colonic tissue of mice with DSS-induced colitis. STING, TBK1, and IRF3 were phosphorylated in these mice, with higher levels of phosphorylation of these proteins observed in the DMXAA group compared with DSS group. In the mice treated with ANP and DMXAA, the expression of cGAS and phosphorylated STING, TBK1, and IRF3 was distinctly reduced, indicating a suppressive function of ANP on the STING pathway. In our immunofluorescence assay, E-Cadherin was used to label CECs in the mouse colon, and the phosphorylation of TBK1 protein was detected. The level of phosphorylated TBK1 in mice with colitis was increased in DMXAA group, and ANP inhibited phosphorylation of TBK1 compared with DMXAA group (Figure 7G).

The tight junction proteins ZO-1 and occludin play a vital role in regulating epithelial permeability, and thus in maintaining the integrity of the gut barrier 37. Hu and colleagues indicated the STING signaling pathway contributed to lethal sepsis by facilitating CEC apoptosis and breach of the colonic barrier 38. While we demonstrated that ANP ameliorates colitis via the STING pathway, the underlying mechanism of ANP in the pathogenesis of UC required further exploration. Thus, to analyze whether ANP could improve the integrity of the colonic mucosal barrier via the regulation of tight junction proteins, the levels of ZO-1 and occludin in the colonic tissue were measured using western blotting. The results showed that the levels of both proteins increased in mice with colitis after ANP administration, compared with untreated and DMXAA treated DSS induced colitis mice (Figure 7I). Moreover, ZO-1 was found to be more highly expressed in the colon of ANP-treated mice compared with the DSS group (Figure 7H). Gut barrier function was further evaluated using an in vivo colonic permeability test with FD4, which can penetrate the bloodstream via impaired intestinal tissue. As shown in Figure 7J, DMXAA treatment in mice with colitis resulted in a significant increase in serum levels of FD4, indicating permeability; this effect was reversed with ANP treatment. Our results showed that ANP could alleviate DSS-induced colonic barrier damage via the STING pathway in mice with colitis.

## Discussion

We hypothesized that ANP may improve colitis by regulating water/salt balance and exerting immunoreactive effects. NPR-A increased the synthesis of cGMP and activated cGMP effector molecules, resulting in a series of physiological and pathological changes, including regulation of cell growth, proliferation, apoptosis, and inflammation 39. Pharmacological intervention to reduce NPR-A activity was able to reduce inflammation in a mouse model of allergic asthma 40. NPR-C has high affinity with ANP, and up-regulates cAMP expression by regulating the activity of adenylate cyclase (AC). NPR-C can also eliminate natriuretic peptides in the circulation or extracellular environment via receptor-mediated internalization and degradation; thus, it is also called the 'elimination receptor' 41. We detected the expression of ANP, NPR-A and NPR-C in murine lymphoid organs and colonic tissues; our results indicated that the ANP and its receptors was involved in colonic immune-related diseases. Next, we collected clinical samples from people with UC and mice with DSS-induced colitis, and found that the expression levels of ANP, NPR-A, and NPR-C were reduced, demonstrating that the natriuretic peptide axis was involved in the occurrence and development of UC. However, the correlation between serum ANP and CRP/ESR/NE indicated that serum ANP level was not related to disease activity. We speculated that UC was a relatively chronic and persistent disease. ANP was found to exert biological effects by binding to its receptors. As disease activity changes, the expression of ANP receptors on target cells may also gradually change, which may explain the lack of variation in serum ANP levels. Different UC medications may have an impact on the expression of serum ANP and colonic STING pathways, but there is currently a lack of research on this topic. It is therefore necessary to collect more clinical samples for comparative analysis in future research and explore the effects of the medications on colitis in DSS-induced mice. ANP is secreted from the atrium into the circulation in response to atrial stretch and extracellular signals, including angiotensin, catecholamines, and angiopressins; the symptoms of colitis, such as intestinal bleeding and diarrhea, may affect blood vessel capacity and thus influence the secretion of ANP. The serum ANP level affects the expression of NPR-A on the target cells. We also found that NPR-A was expressed in the colonic epithelial layer, suggesting that ANP plays a role in colitis by targeting the colonic epithelial layer. The fact that prazosin did not relieve colonic inflammation in mice indicates that ANP does not exert anti-inflammatory effects when serum adrenaline levels are reduced; in light of this, we explored the potential anti-inflammatory mechanism of ANP.

One recent study found that the symptoms of colitis in mice could be alleviated by feeding them with ANP-secreting *Saccharomyces boulardii*, indicating the anti-inflammatory effect of ANP in colitis 42. In line with this, as demonstrated in our results, ANP effectively alleviated colonic inflammation in DSS-induced acute colitis in mice. We also showed that ANP reduced the expression of the STING-related inflammatory cytokines *IFN-α* and *IFN-β*, indicating the involvement of the STING pathway in the mechanism of ANP-induced symptom reduction.

The STING pathway has previously been shown to mediate autoimmune diseases in both mice and humans 17. cGAS cannot distinguish between pathogen-derived dsDNA and endogenic dsDNA; thus the cGAS pathway was activated with dsDNA accumulation in mutant cells, leading to autoimmune disease 17. Most cells contain DNA, so the cGAS-STING pathway plays a role in many inflammatory diseases, including acute pancreatitis, sepsis, colitis, systemic lupus erythematosus, Sjogren's syndrome, and autoimmune encephalomyelitis 37,38,43-46. According to previous research, opinions on the role of the STING pathway in colonic inflammation differ. Gary and colleagues found that STING agonists could significantly aggravate DSS-induced colonic damage and inflammation in mice, while in STING knockout mice, the severity of the disease was found to be reduced 37. However, another study found that lack of the STING gene resulted in greater colonic inflammation and higher risks of intestinal infection in mice 22. Therefore, in this study, we measured STING pathway activation in colonic tissue samples from people with UC and found that phosphorylation of STING pathway components increased, indicating STING pathway activation in these people, consistent with previous results reported by Zhao et al. 47. This confirmed that the STING pathway is involved in the occurrence and development of UC.

ANP binds to its receptors NPR-A and NPR-C to regulate the content of GTP and ATP in cells. When ANP binds to the NPR-A receptor, the conformation of NPR-A changes, and signals are transmitted through the cell membrane, so that guanylate cyclase (GC) forms a 'tight' association. GC catalyzes the production of GTP from cyclic GMP (cGMP) 48. When infection, stress, or cell damage occurs, cGAS recognizes pathogens in the cytoplasm, and uses ATP and GTP to produce the second messenger cyclic GMP-AMP (cGAMP). cGAMP transmits a signal to the STING protein located on the endoplasmic reticulum. Based on the above theoretical basis and the phenomenon that ANP regulates STING pathway activation, we explored the possibility of a direct regulatory relationship between cGAS and NPR-A. Our immunofluorescence assay results showed the co-localization of cGAS and NPR-A, and our co-immunoprecipitation analysis indicated an interaction between cGAS and NPR-A. The cGAS and NPR-A proteins interacted more obviously in mice treated with AMP and DMXAA, providing specific and substantial evidence of ANP regulation of the STING pathway.

Our study is the first to show the inhibitory effect of ANP on the STING pathway in colitis. In a study of septic mice, it was found that the STING pathway was activated, and the destruction of the colonic barrier was increased 38. Activation of the STING pathway was a pivotal step in the development of sepsis via promoting colonic inflammation and destroying the gut barrier. In combination with our other findings, ANP receptors were found to be expressed in CECs, and the level of these receptors was decreased in CECs of mice with DSS-induced colitis, and the expression of IFN-α was also increased in these mice. CEC was the main form of the colonic barrier. We inferred that ANP is able to repair colonic barrier functional damage caused by the activation of the STING pathway in mice with DSS-induced colitis. The detection of ZO-1, occludin, and murine FD4 corroborated this inference.

Recent studies have found that STING coordinates stress-mediated ER autophagy by sensing microbial viability 49. In the case of CEC autophagic defects, transient ER stress activates IFN-I signal transmission in intestinal organoids via the cGAS-STING pathway. The findings from this study support a role for ANP in alleviating ER stress and stimulating ER-dependent apoptosis 50. The presence of the autophagy marker LC3 and the lipidation of LC3-I into LC3-II are signs of autophagy. The formation of autophagosomes involves a ubiquitin-like conjugation system, in which Atg12 covalently binds to Atg5 and targets autophagic vesicles. Beclin1 is a key protein in in autophagy, and its overexpression can stimulate autophagy. In the process of autophagic lysosome degradation, P62 binds to the substrate, which is degraded by proteolytic enzymes. BiP is a molecular chaperone of the ER, which helps proteins fold correctly. If protein folding in the ER is disturbed, BiP synthesis increases. When ER stress occurs, the α subunit of eukaryotic initiation factor 2 (eIF2) is phosphorylated at Ser51, stabilizing the eIF2-GDP-eIF2B complex and inhibiting the turnover of eIF2B. In the early stage of ER stress, SAPK/JNK is activated by its kinase activity, inducing the formation of autophagosomes. The results of the above indicators of autophagy and ER stress not only verified the increase in ER induced autophagy in CECs when the STING pathway was activated but also showed that ANP inhibited a series of negative effects caused by the activation of the STING pathway.

This is the first study of the role of ANP in a DSS-induced colitis model, and the first to explore the association between ANP and STING signaling. We showed that ANP is potentially involved in DSS-induced colitis, and could alleviate colitis by inhibiting the STING pathway resulting in gut barrier repair. We reached the following conclusions: (1) the ANP and its receptors and the STING pathway participate in the pathogenesis of UC in humans and DSS-induced acute colitis in mice; (2) ANP alleviates colonic inflammation via inhibiting the STING pathway; (3) ANP improves colonic barrier function and suppresses ER stress-induced autophagy via the STING pathway; (4) cGAS interacts with the ANP receptor NPR-A.

Our data demonstrate that STING induces colonic inflammation by damaging the gut barrier, while ANP alleviates these effects by inhibiting the STING pathway. Therefore, ANP could be used as a pharmacologic inhibitor of STING signaling, targeting proinflammatory cytokines and regulating gut barrier dysfunction and ER stress-induced autophagy in UC.

## Figures and Tables

**Table 1:** Primers sequences for RT-qPCR.

Gene	Forward	Reverse
Human GAPDH	5′-GGAGCGAGATCCCTCCAAAAT-3′	5′-GGCTGTTGTCATACTTCTCATGG-3′
Human NPPA	5′-TGCAGCTTCCTGTCAACACT-3′	5′-AGAGGCGAGGAAGTCACCAT-3′
Huma NPR-A	5′-CAACCTCGTGGCTGTGAAAC-3′	5′-TCCCAAAGCTGTATACGTCACC-3′
Human NPR-C	5′-GAGGGAGATGCACCGTCAAT-3′	5′-AAGTGAGCACCAGCAGAGAC-3′
Human cGAS	5′-GGTTTCCAAGAAGAAACATGGC-3′	5′-GGGTTCTGGGTACATACGTGAA-3′
Human STING	5′-ACCCCCTTGCAGACTTTGTT-3′	5′-ATCTGCAGGTTCCTGGTAGG-3′
Human TBK1	5′-GACCCGGCTGGTATAACAAGA-3′	5′-TGAACATCCACTGGACGAAGG-3′
Human IRF3	5′-CTGGGGCCCTTCATTGTAGAT-3′	5′-GGCACAACCTTGACCATCAC-3′
Human IFN-α	5′-GGAGGAGTTTGATGGCAACC-3′	5′-ATCCCAAGCAGCAGATGAAT-3′
Human IFN-β	5′-CTTGGATTCCTACAAAGAAGCAGC-3′	5′-TCCTCCTTCTGGAACTGCTGCA-3′
Human Atg5	5′-GACCTTCTGCACTGTCCATCT-3′	5′-GCAATCCCATCCAGAGTTGC-3′
Human Atg7	5′-TGGTTACAAGCTTGGCTGCT-3′	5′-TCAAGAACCTGGTGAGGCAC-3′
Human Atg12	5′-TGCTAAGGGAAAGCTAAAGGCA-3′	5′-GTTCGCTCTACTGCCCACTT-3′
Mouse β-actin	5′-GTTGGAGCAAACATCCCCCA-3′	5′-CGCGACCATCCTCCTCTTAG-3′
Mouse NPPA	5′-TCGGAGCCTACGAAGATCCA-3′	5′-ACACACCACAAGGGCTTAGG-3′
Mouse NPR-A	5′-TCTGGAGGAGAAGCGCAAAG-3′	5′-TCTGGAGGAGAAGCGCAAAG-3′
Mouse NPR-C	5′-AGAGCTCCACCTGCTATCCA-3′	5′-CCATAGGCCTGCAGGAAACA-3′
Mouse TNF-α	5′-CCTGTAGCCCACGTCGTAG-3′	5′-GGGAGTAGACAAGGTACAACCC-3′
Mouse IL-1β	5′-CCTCGTGCTGTCGGACCCATA-3′	5′-CAGGCTTGTGCTCTGCTTGTGA-3′
Mouse IL-6	5′-TAGTCCTTCCTACCCCAATTTCC-3′	5′-TTGGTCCTTAGCCACTCCTTC-3′
Mouse IFN-α	5′-GCTAGGCTCTGTGCTTTCCT-3′	5′-TCCTGCGGGAATCCAAAGTC-3′
Mouse IFN-β	5′-AGCACTGGGTGGAATGAGAC-3′	5′-GAGTCCGCCTCTGATGCTTA-3′

**Table 2:** Clinical features of the participants

	Biopsy samples for PCR			Biopsy samples for western blot			Blood samples for ELISA		
	Ctrl	UC in activity	UC in remission	Ctrl	UC in activity	UC in remission	Ctrl	UC in activity	UC in remission
Number	45	15	9	6	6	6	27	12	11
Age	36 (23-61)	42 (20-62)	44 (23-58)	34 (24-55)	45 (25-60)	42 (26-58)	34 (22-59)	43 (22-61)	47 (28-58)
Sex (Male/Female)	25/20	9/6	5/4	3/3	4/2	3/3	15/12	8/4	5/6
Disease extent									
Proctitis		2	1		1	0		1	1
Left sided		7	3		3	1		7	3
Pancolitis		6	5		2	0		4	2
Current therapy									
5-Aminosalicylates		13	4		5	2		8	4
Steroids		9	1		1	1		5	0
Azathioprine		1	0		0	0		1	0
Biologics		0	0		1	0		0	0
No treatment		0	4		0	3		0	7

## Abbreviations

AC: adenylate cyclase
ANP: Atrial Natriuretic Peptide
ATP: adenosine triphosphate
CD: Crohn's disease
CDNs: cyclic dinucleotides
CEC: colonic epithelial cell
cGAMP: cyclic GMP-AMP
cGAS: cyclic GMP-AMP synthase
cGMP: cyclic GMP
Co-IP: Co-immunoprecipitation
CRP: C-reactive protein
DAI: disease activity inde
DSS: dextran sulphate sodium
ELISA: Enzyme linked immunosorbent assay
ER: endoplasmic reticulum
ESR: erythrocyte sedimentation rate
FD4: Fluorescein isothiocyanate-dextran 4 kD
GC: guanylate cyclase.GTP, guanosine diphosphate
H&E: hematoxylin and eosin
IBD: inflammatory bowel disease
IFN-I: type I interferon
TNF: tumor necrosis factor
IRF-3: interferon regulatory factor 3
LC3: light chain 3
NE: neutrophilicgranulocyte
STING: stimulator of interferons gene
TBK1: TANK-binding kinase 1
UC: ulcerative colitis
